# Empowering analysts to undertake ethically and legally appropriate public good research.

**DOI:** 10.23889/ijpds.v7i3.2021

**Published:** 2022-08-25

**Authors:** Lily O'Flynn

**Affiliations:** 1 UK Statistics Authority

## Objectives

This session will set out the ethical and legal obligations on researchers when collecting, accessing and sharing data, explain why these are important and introduce researchers to frameworks designed to support them in meeting these obligations in an efficient manner.

## Approach

Research governance requirements are sometimes viewed by analysts as introducing friction into the data access process. This session will take the high level ethical and legal principles set out in the Research Code of Practice and Accreditation Criteria that researchers are often required to adhere to and translate them into relatable frameworks that can be easily applied to statistical research projects. It will overview the open-source support available across the research community for analysts needing to overcome identified ethical and legal barriers to enable efficient access to data for public good research that maintains public trust.

## Results

Issues with the timelines involved with access to data are widely cited. Over the last year, the frameworks and associated user support overviewed in this session have enabled over 250 research projects to efficiently move forward in an ethically and legally appropriate way, with users across government, academia and the private sector and representation across all disciplines. We have found that helping researchers understand why such research governance requirements are in place and providing recognised and user-focused solutions for meeting such requirements speeds up the data access process. Researchers that attend this session will understand how the Research Code of Practice and Accreditation Criteria is applied to research, how to demonstrate compliance with these requirements, and support available for achieving this.

## Conclusion

This session aims to equip analysts with tools and support that enable them to ensure that their research is ethically and legally appropriate by design, and therefore empower the research community minimise ethical and legal barriers which may prevent or prolong access to data for public good research.

